# Murine Breast Cancer Radiosensitization Using Oxygen Microbubbles and Metformin: Vessels Are the Key

**DOI:** 10.3390/ijms241512156

**Published:** 2023-07-29

**Authors:** Agnieszka Drzał, Gabriela Dziurman, Paweł Hoła, Jakub Lechowski, Anthony Delalande, Jan Swakoń, Chantal Pichon, Martyna Elas

**Affiliations:** 1 Faculty of Biochemistry, Biophysics and Biotechnology, Department of Biophysics and Cancer Biology, Jagiellonian University, 30-387 Krakow, Poland; agnieszka.drzal@uj.edu.pl (A.D.); gabriela.dziurman@doctoral.uj.edu.pl (G.D.); pawel.hola@student.uj.edu.pl (P.H.); jakub.lechowski@student.uj.edu.pl (J.L.); 2 Doctoral School of Exact and Natural Sciences, Faculty of Biochemistry, Biophysics and Biotechnology, Department of Biophysics and Cancer Biology, Jagiellonian University, 30-387 Krakow, Poland; 3 UFR Sciences and Techniques, University of Orleans, 45067 Orleans, France; anthony.delalande@cnrs.fr (A.D.); chantal.pichon@cnrs.fr (C.P.); 4 Center for Molecular Biophysics, CNRS Orleans, 45071 Orleans, France; 5 Institute of Nuclear Physics, Polish Academy of Sciences, 31-342 Krakow, Poland; jan.swakon@ifj.edu.pl; 6 Institut Universitaire de France, 75231 Paris, France

**Keywords:** oxygen microbubbles, metformin, hypoxia, radiotherapy, breast cancer

## Abstract

Radiotherapy is a cornerstone of cancer treatment, but tumor hypoxia and resistance to radiation remain significant challenges. Vascular normalization has emerged as a strategy to improve oxygenation and enhance therapeutic outcomes. In this study, we examine the radiosensitization potential of vascular normalization using metformin, a widely used anti-diabetic drug, and oxygen microbubbles (OMBs). We investigated the synergistic action of metformin and OMBs and the impact of this therapeutic combination on the vasculature, oxygenation, invasiveness, and radiosensitivity of murine 4T1 breast cancer. We employed in vivo Doppler ultrasonographic imaging for vasculature analysis, electron paramagnetic resonance oximetry, and immunohistochemical assessment of microvessels, perfusion, and invasiveness markers. Our findings demonstrate that both two-week metformin therapy and oxygen microbubble treatment normalize abnormal cancer vasculature. The combination of metformin and OMB yielded more pronounced and sustained effects than either treatment alone. The investigated therapy protocols led to nearly twice the radiosensitivity of 4T1 tumors; however, no significant differences in radiosensitivity were observed between the various treatment groups. Despite these improvements, resistance to treatment inevitably emerged, leading to the recurrence of hypoxia and an increased incidence of metastasis.

## 1. Introduction

Hypoxia (low tissue oxygenation) is common in solid tumors, including breast, lung, prostate, and brain cancers [1]. Several factors contribute to the development of hypoxia in tumors [2]. The rapid proliferation of cancer cells leads to an increased need for oxygen and nutrients, outpacing the formation of new blood vessels to supply the tumor. Moreover, tumor blood vessels’ irregular and abnormal architecture results in inefficient oxygen delivery [3]. Furthermore, cancer cells often have altered metabolism, leading to increased consumption of oxygen [4]. Hypoxia in the tumor microenvironment has significant implications for cancer progression and resistance to treatment. It influences various aspects of tumor biology, including angiogenesis (formation of new blood vessels), metastasis (spread of cancer cells), immunosuppression, and resistance to chemotherapy and radiation therapy [5,6,7].

Given the importance of hypoxia in cancer biology, targeting hypoxic regions within tumors has become an active area of research [8,9,10]. Strategies under investigation include, among others, the use of drugs that sensitize hypoxic cells to radiotherapy [11,12,13], agents that inhibit angiogenesis [14,15], and novel therapies that directly target the hypoxia signaling pathways [16,17,18]. An innovative approach to the problem of tumor hypoxia is the use of ultrasound-sensitive oxygen microbubbles (OMBs) that release oxygen locally into tumor tissue [19,20,21,22]. Their use in cancer therapy has been explored for several years. Recently, our group has shown that anionic pegylated OMBs composed of distearoylphosphatidylcholine (DSPC) and PEGylated 1,2-distearoyl-sn-glycero-3-phosphorylethanolamine (DSPE-PEG2000) efficiently increase tumor oxygenation [23,24].

Every pharmacological way to improve tumor tissue oxygenation encounters difficulties related to the single most basic cause of hypoxia—pathological and non-functional tumor vascularization that impedes the delivery of therapeutic substances [25]. One way of dealing with abnormal tumor vasculature is the use of therapies leading to a transient “therapeutic window” characterized by normalization of vessels, improvement of oxygenation, and decrease of the metastatic potential of the tumor [26]. These include antiangiogenic therapies and therapies aiming to regulate the oxygen partial pressure in the tumor. The time window of normalization is limited and depends on factors such as the concentration/type of antiangiogenic agent or cancer type that determines the acquisition of resistance to therapy [27].

Metformin is an orally administered biguanide commonly prescribed for treating type 2 diabetes [28]. Most of its pharmacological effects are due to the activation of 5’AMP activated kinase (AMPK), which acts in the cell as an energy sensor. Activation of AMPK blocks anabolic pathways and activates catabolic pathways, thereby reducing cellular oxygen consumption [29]. Numerous preclinical studies have confirmed its antitumor properties [30]. Metformin inhibits the growth of multiple tumor lines, including breast cancer, blocks cellular transformation in an inducible model system, and exhibits anti-tumor activity in murine xenografts [31,32,33,34]. Moreover, due to metformin’s activity as a respiration inhibitor, a significant increase in tumor oxygenation and radiosensitization was observed in multiple cancer types [35,36]. It was proposed that metformin’s antiangiogenic effect is partly responsible for these effects [37,38].

We studied the synergistic action of metformin and oxygen microbubbles and the impact of this drug combination on tumor vasculature, oxygenation, aggressiveness, and radiotherapy effectiveness in a murine 4T1 breast tumor.

## 2. Results

**Figure 1:**
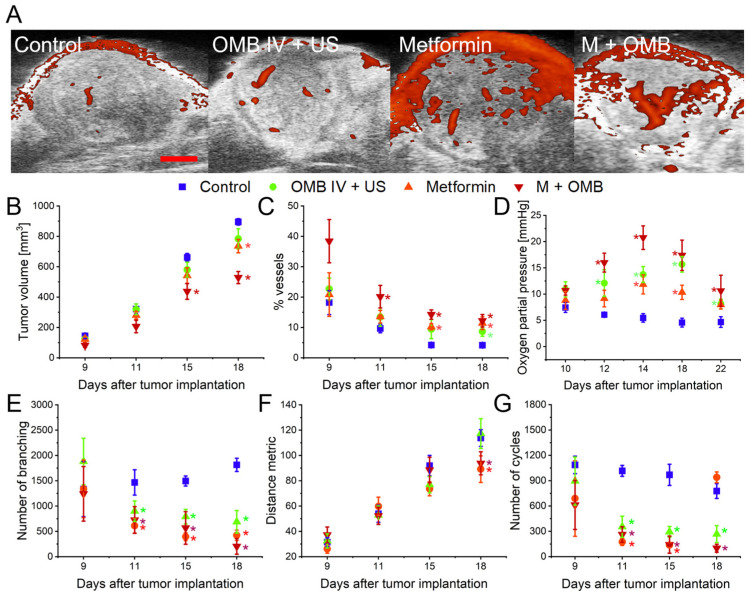
Impact of therapy on tumor vasculature and oxygenation. (**A**) Representative Power Doppler cross-sections of a tumor with tumor vasculature (red) from each experimental group. The red scale bar in the first image represents 1 cm. Numerical analysis of imaging data provided mean values of tumor volume (**B**), and vasculature content (**C**) throughout therapy. Electron paramagnetic resonance oximetry allowed oxygenation monitoring during therapy (**D**). Vascular tree analysis of Power Doppler images showed the effect of treatments on branching (**E**), tortuosity (**F**), and the amount of closed vascular loops (**G**) in tumor vasculature. * *p* < 0.05 relative to the control. N = 10 mice in each group, total N = 40. The error bars represent SEMs for the data of each group.

Ultrasound Power Doppler imaging demonstrated growth kinetic and vascular changes due to treatment with metformin, oxygen microbubbles, and their combination (Figure 1). Treatment with combination therapy and metformin alone led to significantly slower tumor growth. Moreover, tumor vasculature was also affected. All therapies resulted in more vascularized tumors, especially in the center (Figure 1A). We performed an analysis of the vascular structure using the vascular trees approach to see the signs of vascular normalization. Starting on day 11 of therapy, all therapeutic protocols led to lower branching and number of cycles (closed vascular structures characteristic of tumor vasculature arising from hectic angiogenesis [39]), which may indicate a normalization of tumor vasculature (Figure 1E–G). However, on the 18th day of tumor growth, a sudden increase in this parameter was visible in a group treated with metformin only. Combination therapy and metformin-treated tumors were also characterized by a decreased distance metric, showing that the vessels were less torturous. However, a significant difference was visible only on the 18th day of tumor growth.

Besides ultrasound, we also performed electron paramagnetic resonance (EPR) oximetry with an implantable Oxychip probe to track changes in tumor oxygenation throughout the therapy. Importantly, EPR measurements were not performed on a day of OMB injections (days 6th, 13th, and 20th of tumor growth) to see only the long-term results of treatment. All protocols led to significantly higher oxygenation of a tumor starting on day 12, and the effect was the strongest with combination therapy. The highest effect was visible on day 14; therefore, this day was selected as the one in which normalization of tumor vasculature is the most probable, and was analyzed in detail in the second cohort of animals.

On the 22nd day, all animals were sacrificed, and tumors and lungs were analyzed. Combination therapy and metformin-treated tumors were significantly smaller (0.84 g and 0.87 g, respectively, vs. 1.25 g for control, *p* = 0.03), however, they also had more metastatic lesions in the lungs. The number of metastases assessed macroscopically after lung isolation at necropsy was 13, 19, 22, and 32 (*p* = 0.02 vs. control) in the control, OMB IV + US, metformin, and OMB + M groups, respectively.

**Figure 2:**
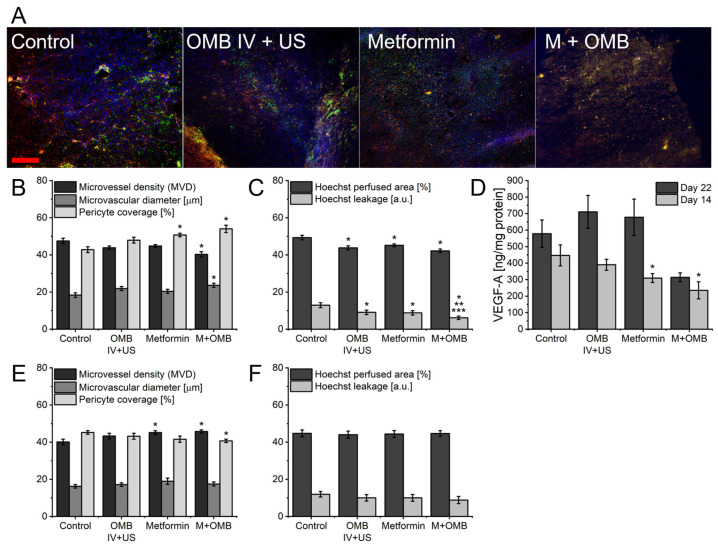
Impact of therapy on tumor microvasculature structure, perfusion, and vascular endothelial growth factor (VEGF) concentration. (**A**) Representative fluorescent images of 14-day tumor slices from experimental groups with stained vascular endothelial cells (CD31, red), pericytes (NG2, green), and Hoechst 33342 dye perfusion (blue). The red scale bar in the first image represents 100 μm. Mean values of parameters determined from photos of tumor slices from the described experimental groups: (**B**,**E**) microvessel density, microvessel diameter, and vessel coverage with pericytes and (**C**,**F**) Hoechst perfused area and Hoechst dye leakage. Graphs (**B**,**C**) represent results from tumors collected on day 14th. Graphs (**E**,**F**) represent results from tumors collected on the 22nd day. (**D**) VEGF-A concentration in tumors from the described experimental groups collected at the described time points measured with ELISA. * *p* < 0.05 relative to the control, ** *p* < 0.05 relative to the OMB IV + US group, *** *p* < 0.05 relative to the metformin group. N = 10 mice in each group, total N = 40. The error bars represent SEMs for the data of each group.

Tumors collected during necropsy from animals sacrificed on the 22nd and 14th days were used to analyze microvasculature structure, perfusion, and vascular endothelial growth factor (VEGF) concentration (Figure 2). At the end of the experiment, on day 22, no apparent signs of vascular normalization were visible. Tumors treated with a combination of metformin and oxygen microbubbles had slightly higher microvessel density (MVD) and lower pericyte coverage than control, however, these changes were so small that no differences in vessel perfusion were discovered. Similarly, metformin treatment led to a slightly higher MVD with no differences in other parameters analyzed. The same was apparent in the level of VEGF-A in tumor homogenates, showing a rather insignificant increase after treatment with a single agent and a slight decrease with their combination. On the contrary, tumors collected on day 14 (after 2 doses of OMB and 14 doses of metformin) showed clear characteristics of vascular normalization (Figure 2B,C). Combination therapy led to a slight decrease in microvessel density, increased vessel diameter, and pericyte coverage. Additionally, vessels were less permeable compared to control and single treatment groups as shown by decreased Hoechst leakage. The same permeability reduction was also observed in tumors treated with oxygen microbubbles alone and metformin alone; however, no significant changes in microvessel structure were detected apart from an increase in the coverage of pericytes in the metformin-treated group. Moreover, the normalization process at day 14 was also confirmed by a slight, but significant decrease in VEGF-A concentration in tumor tissue in both metformin and combination therapy.

**Figure 3:**
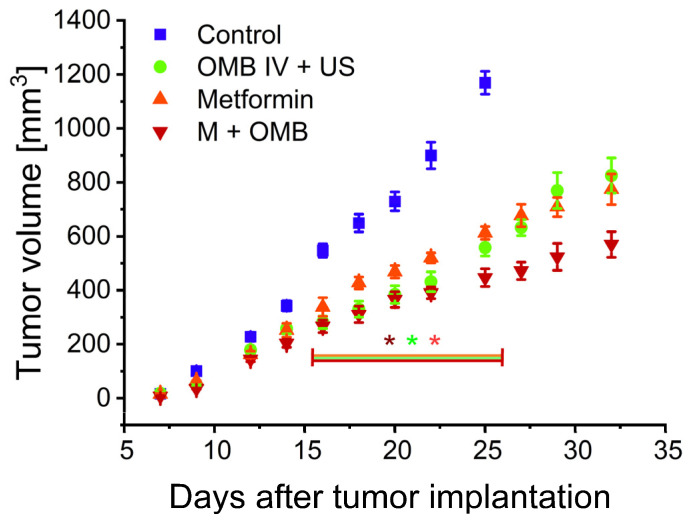
Influence of the therapy on the effectiveness of a single radiation dose. Growth kinetics of 4T1 tumors treated with oxygen microbubbles, metformin, or their combination and radiation. The control contained animals treated only with radiotherapy. * *p* < 0.05 relative to the control for all groups between days 16 and 25. N = 10 in each group, total N = 40. The error bars represent SEMs for the data of each group.

Oxygen exhibits potent radiosensitizing properties, but its impact can only be observed when it is available in tumor tissue during radiotherapy. We assessed the efficacy of all three therapy protocols in enhancing the tumor response to a single 12 Gy gamma radiation dose; that dose alone is insufficient for achieving complete tumor control. All animals were irradiated on the 14th day of the studied therapies. The growth kinetics of the treated tumors are presented in Figure 3. All three protocols were equally effective and led to almost two times slower tumor growth after radiotherapy.

The animals were sacrificed when the tumor reached the volume of 1000 mm^3^ or apparent signs of decreased welfare were observed. All animals from the control group were euthanized due to tumor size. On the other hand, in treated groups with smaller tumors (mean tumor weight was 1.43 g, 1.25 g, and 0.76 g for the OMB IV, metformin, and OMB + M groups, respectively, versus 1.87 g for control) most of the animals were sacrificed due to breathing problems and weight loss, indicators of metastases in the lungs. The number of metastases assessed macroscopically after lung isolation at necropsy was 23, 27, 34, and 45 (*p* = 0.05 vs. control) in the control, OMB IV + US, metformin, and OMB + M groups, respectively.

**Figure 4:**
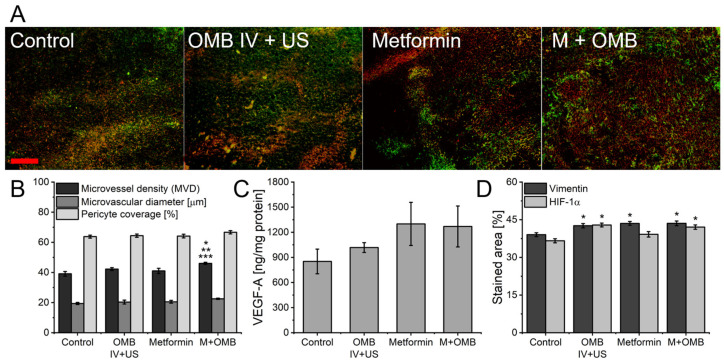
Impact of the therapy and 12 Gy radiation on tumor microvasculature structure, vascular endothelial growth factor (VEGF) concentration, hypoxia, and invasiveness markers. Tumors were collected between days 25 and 33. (**A**) Representative fluorescent images of tumor slices stained for vimentin (red), and HIF-1α (green). The red scale bar in the first image represents 100 μm. (**B**) Mean values of parameters determined from photos of tumor slices from the described experimental groups: microvessel density, microvessel diameter, and vessel coverage with pericytes. (**C**) VEGF-A concentration in tumors from the described experimental groups measured with ELISA. (**D**) Mean values of the percent of vimentin and hypoxia-inducible factor 1α stained area determined from photos of tumor slices from the described experimental groups. * *p* < 0.05 relative to the control, ** *p* < 0.05 relative to the OMB IV + US group, *** *p* < 0.05 relative to the metformin group. N = 10 mice in each group, total N = 40. The error bars represent SEMs for the data for each group.

Tumors collected during the necropsy of animals treated with proposed therapies and radiation therapy were used to assess the structure of the microvasculature, perfusion, and VEGF concentration (Figure 4), similar to tumors collected in previous animal cohorts. Due to the increased number of metastatic lesions in the lungs of treated mice, hypoxia-inducible factor 1α (HIF-1α), a marker of hypoxia, and vimentin, upregulated after epithelial–mesenchymal transition (EMT) crucial in the process of metastasis, were also assessed. Analysis of microvessels revealed increased MVD in tumors treated with combination therapy and radiotherapy compared not only to control but also to tumors collected from mice treated with each therapy separately. The most pronounced differences occurred on the level of HIF-1α and vimentin. All treatment groups had significantly higher expression of vimentin. Moreover, both, treatment with oxygen microbubbles, and their combination with metformin led to an increased amount of HIF-1α.

## 3. Discussion

Vascular normalization is a concept in cancer therapy that refers to the process of restoring abnormal blood vessels in the tumor to a more normal state. In cancer, tumor blood vessels are often structurally and functionally disturbed, leading to inefficient blood flow, poor oxygenation, and impaired drug delivery to the tumor. Vascular normalization aims to modulate vessels to be more uniform in structure, less tortuous, and less leaky, thus improving tumor perfusion and creating a more favorable environment for cancer treatments such as chemotherapy or radiation therapy [40].

While metformin is known primarily for its role in diabetes management, there is emerging research exploring its potential effects on tumor vasculature and vascular normalization in cancer therapy [41,42]. Some studies have suggested that metformin may contribute to vascular normalization through various mechanisms, such as inhibition of angiogenesis, modulation of endothelial cell function, and antifibrotic effects [37,43]. It was shown that treatment with metformin leads to inhibition of endothelial cell migration [44], reduction of VEGF expression [45], decreased expression of the key in angiogenesis metalloproteinases 2 and 9 [46], and lowers the activation of HIF-1α [47]. In addition to its direct effects on tumor blood vessels, metformin has also been shown to have indirect effects on the tumor microenvironment. It can modulate the immune response, reduce inflammation, and affect tumor metabolism, all of which can contribute to vascular normalization [48,49,50,51]. It has been demonstrated that metformin significantly affects the regulation of programmed death-ligand 1 expression in both the tumor cell membrane and intra-cellularly [52,53]. Additionally, the disruption of mitochondrial function induced by metformin leads to a decrease in the expression of the multidrug resistance protein 1, potentially enhancing the accumulation of drugs within tumor cells [54].

Oxygen microbubbles have been studied for several years mostly as a means to increase tumor oxygenation for enhanced radio- or chemotherapy. After being destroyed with a high-power ultrasound pulse, they cause mechanical and oxygen-dependent responses in tumor tissue and cancerous microvasculature [20,22,23,24]. It was proposed that inhibition of tumor angiogenesis and progression can be achieved by reducing the expression of HIF-1α, a downstream factor that is suppressed when oxygen levels are increased. Consequently, this leads to the inhibition of VEGF, limiting the formation of new blood vessels within the tumor. Ho et al. showed that a single injection of oxygen microbubbles leads to a 6-day vascular normalization window characterized by increased coverage with pericytes of tumor vessels, Hoechst penetration, and intratumoral accumulation of doxorubicin [19].

Adaptive resistance to antiangiogenic therapy is characterized by activation/enhancement of alternative proangiogenic signaling pathways, bone marrow-derived cells (BMDCs) recruitment, and alternative neovascularization [55,56,57]. Additionally, due to the activation of receptor tyrosine kinase (c-MET), the infiltration of neutrophils by S100A4 and increased expression of invasion-promoting integrin β1 in cancer cells is observed [58,59,60]. Similar complications are also observed with therapies that regulate oxygen partial pressure in the tumor, as most of these mechanisms are activated by hypoxia immediately after the normalization period.

Ultrasound results (Figure 1) showed a significant decrease in tumor growth rate and an increase in the vascular content of tumors treated with metformin and its combination with oxygen microbubbles. It has been shown in several cancer models that metformin, by activation of AMP-activated protein kinase (AMPK), inhibits cancer cell growth, as it regulates multiple downstream signaling pathways involved in cell metabolism, proliferation, and survival [30,61,62]. Moreover, metformin can suppress the mammalian target of rapamycin (mTOR) pathway, which is crucial for cell growth and proliferation. By inhibiting mTOR, metformin can hinder tumor cell division and survival [63].

Several preclinical studies using animal models have shown that metformin can improve tumor vascular function [38]. Similarly, it has been found to reduce the abnormal structure of tumor blood vessels and increase the density of functional blood vessels within tumors [64]. Power Doppler can image only functional vessels; slow or turbulent flow and chaotic vessel structure make imaging difficult, and not all vessels are detected using this method. The increased number of visible vessels could be the result of higher vascularity (not a sign of normalization) or better functionality of imaged vessels. Vascular tree analysis confirmed that metformin-treated tumors had less branched out and torturous vessels. Moreover, tumor vessels formed less closed vascular structures characteristic of tumor vasculature arising from fast angiogenesis. However, on the 18th day of tumor growth, a sudden increase in this parameter was visible in a group treated only with metformin, which can be a sign of resistance acquisition. Treatment with oxygen microbubbles also leads to higher vascularization in tumors, however, the characteristics of this process are slightly different from those exerted by metformin. The vessels remained as winding as in the control but had fewer branching and vascular loops. These characteristics may be the result of different treatment schemes, as oxygen microbubbles were administered once a week, resulting in subsequent doses being administered long after the normalization window created by the previous injection ended.

Metformin, acting as an inhibitor of the NADH dehydrogenase complex, inhibits cellular respiration, reducing oxygen consumption and thus increasing tissue oxygenation. This effect was associated with a reduction in HIF-1α and VEGF expression in several cancer models [65,66,67]. Similarly, oxygen microbubbles have been shown to effectively increase tumor oxygenation and exert changes on the level of these key regulators of hypoxia response and angiogenesis [19,22,23,24]. We utilized a sensitive, quantitative, and direct oximetric method, the electron paramagnetic resonance, to measure changes in tumor oxygen partial pressure during proposed therapies [68,69,70,71]. After single probe insertion at the beginning of tumor growth, it allows repetitive measurements of oxygen partial pressure (pO_2_) from the implantation site. We observed the highly hypoxic character of control tumors with oxygenation around 5 mmHg. All treated tumors exhibited higher pO_2_ than the control; however, changes in time differed depending on the experimental group. Tumors treated with oxygen microbubbles exhibited the highest oxygenation on day 18th. Interestingly, oxygen microbubbles were administered on days 6, 13, and 20, so the time point with the highest oxygenation is the one the furthest from injection time. Furthermore, the effect of a single dose of oxygen microbubbles on tumor pO_2_ was shown to be fairly short, lasting no more than 15–20 min [23]. This implies that maybe it is not oxygen itself, but rather molecular responses to its short-term increase and mechanical changes in tumor vasculature due to microbubble cavitation that are responsible for potential vessel normalization. Both metformin-treated groups have a peak oxygenation on day 14 of therapy. Metformin alone led to a moderate increase resulting in stable oxygenation of around 10–15 mmHg throughout the tumor growth. However, in combination with oxygen microbubbles, a clear maximum reaching even 20 mmHg was observed on day 14. Since the Doppler ultrasound results were not straightforward and did not show us a clear normalization window, we decided to pick this time point to test the enhancement of radiotherapy with proposed therapies and look at tumor microvasculature and perfusion.

Vascular normalization is characterized by a lower concentration of VEGF-A, a decrease in microvessel density, permeability, and an increase in its size and pericyte coverage [26,72]. We studied those parameters in tumors collected both on days 22 and 14 (Figure 2). On day 22 only tumors treated with metformin showed any significant differences from control. It had higher MVD and lower pericyte coverage. Moreover, they had slightly higher VEGF-A concentrations. These characteristics led to the conclusion that this time point represents tumors with acquired resistance. The situation was much better in the tumors collected on day 14. Tumors treated with metformin had a higher pericyte coverage, lower permeability, and VEGF-A concentration, but in combination with oxygen microbubbles, all features of vascular normalization were detected. Oxygen microbubbles lead only to a lower permeability of the tumor vessels. This must be connected to repeated dosing, since it was shown that cavitation of MB in tumor vasculature causes permeability to increase 3 h after microbubbles burst [24]. On the other hand, Ho et al. showed normalization after 1 dose of oxygen microbubbles lasting for approx. 6 days and the first time-point studied was 2 days after the OMB injection [19]. Mechanisms of normalization after treatment with oxygen microbubbles and metformin are different, but it is possible to combine them to achieve the strongest effect. Together, ex vivo findings explain the results of in vivo Doppler imaging, i.e., increased tumor vascularization as a result of lower vessel permeability making them more “visible” for functional imaging.

Oxygen plays a critical role in the effectiveness of radiotherapy. The presence of oxygen enhances the damaging effects of radiation on cancer cells. When ionizing radiation interacts with oxygen molecules, it generates reactive oxygen species (ROS) and free radicals that can cause additional DNA damage and oxidative stress within cancer cells [73]. Among many radiosensitizing strategies, normalization of the tumor vasculature was extensively studied with various results [74]. It has been shown that the timing, duration, and specific methods used for vascular normalization, as well as the individual characteristics of tumors, may all influence the outcomes. Moreover, tumor vessel normalization may influence the effectiveness of radiotherapy not only by reversal of the tumor hypoxia but also by alleviating the immunosuppressive environment by promoting enhanced T-cell infiltration and boosting antitumor immune activity [75]. We saw a significant increase in the effectiveness of radiotherapy after each of the proposed therapy protocols without differences between them (Figure 3). We confirmed by EPR oximetry and ex vivo microvascular analysis that the 14th day lies within the normalization window of the therapies; therefore, we are sure that this was a reason for this increased efficacy. Furthermore, we saw that an increase in oxygenation in each group, despite being at different levels, was around or even above 15 mmHg. The relationship between radiosensitivity and oxygenation follows an asymptotic pattern [76]. This means that as the oxygen levels approach and reach a certain point (around 10–15 mmHg), the additional increase in radiosensitivity becomes minimal. Beyond this point, additional increases in oxygenation do not result in a significant additional enhancement of radiosensitivity, which is a reason for the lack of differences between our therapy protocols.

Several studies have investigated the impact of metformin on tumor metastasis, the process by which cancer cells spread from the primary tumor to other parts of the body. While the exact mechanisms are not fully understood, there is evidence to suggest that metformin may have some inhibitory effects on tumor metastasis [77]. Among others, metformin has been shown to inhibit the growth and proliferation of various cancer cell lines, suppress their migratory capabilities, and have anti-inflammatory properties, potentially reducing the inflammatory signals within the tumor microenvironment. With and without radiotherapy, we observed an increased metastatic spread of treated tumors to the lung. Significantly more metastatic lesions were visible in groups treated with metformin, but oxygen microbubbles alone also led to an increase in this parameter. The inconsistency of our results may lie in dosing focused on vascular normalization. It is known that after the end of the normalization window in tumor tissue hypoxia returns, with promotion of pathological angiogenesis, and drug resistance, all leading to increased metastasis [57]. Vascular normalization improves the integrity of blood vessels, reducing tumor cell intravasation (entry into the bloodstream) and subsequent metastasis. Again, after the normalization window, the integrity of the vessel is compromised, creating leaky vessels and facilitating the intravasation and dissemination of tumor cells to distant sites (Figure 4). In tumors harvested between days 25 and 33, we observed an increased amount of HIF-1α, being indicative of hypoxia return after the end of the normalization window. Hypoxia has been shown to influence the invasive and migratory behavior of cancer cells by stimulating the epithelial–mesenchymal transition, one of the most crucial steps of the metastatic cascade [78]. EMT is stimulated by the TGF-β master regulator, whose expression is upregulated under hypoxic conditions. TGF-β activates downstream transcription factors such as Smad, Snail, Slug, and Twist, which can also directly interact with HIF due to the presence of HRE in their promoter. An increase in the activation of these transcription factors under the influence of hypoxia has been described [79]. Coexpression of HIF-1α, TWIST, and Snail in primary tumors was also shown to correlate with a worse prognosis in patients [5]. Moreover, vimentin, a marker of epithelial–mesenchymal transition, was also upregulated after the studied therapies.

## 4. Materials and Methods

### 4.1. Microbubbles Formulation and Disruption

A liposomal formulation of DSPC:DSPE-PEG2000 (0.9:0.1 molar ratio) was prepared as previously described [23]. Briefly, 5 mg/mL lipid solution in HEPES buffer (Gibco, Thermo Scientific, Waltham, MA, USA) was freeze-dried and the headspace of the vial was replaced by C_4_F_10_ gas (F2 Chemicals, Lea Town, UK). Before the experiments, the perfluorocarbon was replaced with oxygen, the vial content was resuspended in 500 µL of HEPES buffer and activated by mechanical agitation using a VialMix (Bristol-Myers Squibb, New York, NY, USA) for 45 s. Microbubbles were destroyed using a Vevo Sonigene device (VisualSonics, Toronto, ON, Canada) sending ultrasound at 1 MHz at 2 W/cm^2^ for 1 min. Attention was taken to use microbubbles no later than 30 min after activation.

### 4.2. Animals and Tumor Model

4T1 cells were grown at 37 °C in a humidified atmosphere of 5% CO_2_/95% air in RPMI 1640 (Sigma Aldrich, St. Louis, MO, USA) containing 10% heat-inactivated fetal bovine serum (Gibco, Thermo Scientific, USA) plus penicillin-streptomycin (Sigma Aldrich, USA) under sterile tissue culture conditions.

BALB/cAnNRj female mice at the age of 3 months were originally obtained from the Janvier Labs (Le Genest-Saint-Isle, France). All experimental procedures were approved by the 2nd Local Institutional Animal Care and Use Committee in Cracow (Permission No. 189/2021). The mice were housed under standard laboratory conditions LD:12/12, humidity: 60%, temperature: 23 °C. The standard food diet with free access to drinking water was provided in community cages.

For each mouse, 5 × 10^5^ 4T1 cells, suspended in 50 µL of PBS, were injected into the mammary fat pad. Three separate animal cohorts (impact of therapy on tumor microenvironment, characterization of the normalization window, and radiotherapy enhancement), each consisting of 40 mice, were used with different endpoints: euthanasia after the development of metastases or tumor size greater than 1000 mm^3^ (for cohorts one and three) or at the point of the highest increase in oxygenation (cohort two). Within each cohort, the animals were divided into four experimental groups (N = 10 in each experimental group): (a) metformin-treated animals (350 mg/kg daily since cancer cell inoculation, intraperitoneal injection), (b) oxygen microbubbles-treated animals (OMB IV + US, tumor subjected to ultrasound impulse after intravenous administration of 100 µL of oxygen microbubbles, once a week since day 6 of tumor growth, on day 6, 13 and 20), (c) animals treated with both metformin and oxygen microbubbles with the same treatment schemes as in groups a and b (M + OMB), (d) untreated control.

### 4.3. In Vivo Electron Paramagnetic Resonance (EPR) Measurements

In vivo EPR measurements were performed on an L-band (Bruker Elexsys-II E540, Germany) CW EPR spectrometer using a surface coil (Bruker, Ettlingen, Germany). Anesthesia was induced with 3 vol% isoflurane (Aerrane, Baxter Polska Sp. z o. o., Warszawa, Poland) and then maintained at 1.5–2.0 vol% isoflurane in air, administered at 1.2 L/min through a nose mask. The breathing rate and temperature were monitored.

Spectroscopy mode was used to measure the changes in tumor oxygenation during therapy. Measurements were performed twice a week starting from day 10 of tumor growth. In vivo EPR spectroscopy was performed with the following parameters: microwave power = 10.75 mW; center field = 389 G; modulation frequency = 100 kHz; modulation amplitude = 0.018 G. Oxychip (a kind gift from Prof. Perianiann Kuppusamy, Dartmouth Medical School, Dartmouth, NH, USA) was used as an oxygen-sensitive spin probe [80,81]. A 1 mm long piece of Oxychip was implanted in tumor tissue using a 22G needle 3 days before EPR measurements (on the 7th day of tumor growth). This spin probe coupled with spectroscopic EPR measurements allows us to get information about the mean oxygen concentration (calculated from spectrum line width based on the calibration curve) near the implantation site.

The obtained spectra were analyzed using a script written in the Matlab environment. Briefly, Gauss and Lorentz’s curves were fitted to the probe spectra using the esfit function (part of the EasySpin spectroscopic data analysis package, https://www.easyspin.org/ accessed on 1 June 2023) using the least squares method and the particle swarm algorithm. This made it possible to determine the width of the spectrum without respiratory artifacts. The obtained spectral widths were converted into the values of the partial oxygen pressure by using the calibration curve.

### 4.4. In Vivo Power Doppler Ultrasonographic Imaging

A High-Resolution Ultrasound Imaging System, designed for the examination of small animals (VisualSonics Vevo 2100, Toronto, ON, Canada) with an MS-550D transducer was used for the study. During the imaging process, body temperature was controlled by a heating pad and maintained at 37 °C. Anesthesia was induced by 3 vol% isoflurane (Aerrane, Baxter Polska Sp. z o. o., Warszawa, Poland) and then maintained at 1.5–2.0 vol% isoflurane in the air, delivered at 1.2 L/min via a nose mask.

For the initial confirmation of the tumor, a B-mode ultrasound (grayscale) was performed with a central frequency of 40 MHz. Doppler imaging was used to detect the presence of blood flow and to evaluate the direction and speed of flow in vessels of a diameter larger than 30 µm. Power Doppler (PD) measurements were performed with a central frequency of 32 MHz and pulse repetition frequency (PRF) of 3–4 kHz. 3D images of tumors, each containing about 40 scans per tumor, were obtained using a steady handheld transducer. Measurements were performed twice a week starting from day 9 of tumor growth.

Data analysis was performed in Vevo LAB version 5.6.1. software (VisualSonics, Toronto, ON, Canada). The tumor outline was defined in Power Doppler images by an experienced researcher manually, based on the B-mode tumor image and visible vascular structure. Tumor volume, tumor vasculature volume, and their ratio were calculated. Images with tumor masked were used for further analysis with the use of an in-house script written in Python3. The tumor vasculature was binarized and skeletonized, and after finding line-end-points, the build of a spanning tree was performed with the use of a breadth-first search algorithm [82]. Based on the prepared vascular tree, several morphological characteristics of the cancer vasculature were calculated: number of cycles (closed loops of the vascular network), number of branches, number of vascular trees, and distance metric (the ratio of the length of the vessel to the length of the line drawn from the first element of the tree to the last element; the larger the length metric, the more tortuous the vessel).

### 4.5. Irradiation

Irradiation was performed under general anesthesia. Anesthesia was induced with 3% isoflurane and then maintained at 1.5–2.0% isoflurane in air, administered at 1.2 L/min through a nose mask. The tumor was mechanically retracted away from the peritoneal cavity and secured with tape. The rest of the body of the mouse was covered with lead shielding. Irradiation was carried out on day 14 of tumor growth using the THERATRON 780E radiotherapy unit (produced by MDS Nordion, Ottawa, ON, Canada) equipped with a ^60^Co cobalt gamma ray source. A total dose of 12 Gy was deposited at a rate of 1 Gy/min. The control group was also irradiated.

After the irradiation, animals were monitored every two days. Tumors were measured with a caliper. The animals were sacrificed when the tumors reached a volume of 1000 mm^3^ or signs of decreased well-being (breathing problems, weight loss greater than 20%) were detected.

### 4.6. Histology

Dissected lungs were snap-frozen in Cryomatrix (Thermo Shandon, Waltham, MA, USA), and stored at −80 °C. All samples were cut into 10-μm sections, mounted on glass slides, fixed in cold ethanol for 1 min, and stored in PBS at 4 °C. Standard hematoxylin-eosin-stained sections were evaluated for metastatic lesions under a light microscope at a magnification of 200×.

### 4.7. Vascular Morphology and Perfusion Assessment

The detection of vessels, pericytes, and perfusion was performed using triple immunofluorescence (IF) staining. One minute before dissection, mice received an intravenous injection of Hoechst 33,342 solution (15 mg/kg body weight, Sigma Aldrich, USA). At the selected time point, tumor tissue was harvested, snap-frozen in the Cryomatrix, and stored at −80 °C. All samples were cut into 10-μm sections and mounted on glass slides. The sections were air-dried for 10 min and stored at −20 °C. Immunohistochemical staining was performed with rabbit anti-CD31 and rat anti-NG2 (PA5-16301, MA5-24247, 1:200, Invitrogen, Waltham, MA, USA) antibodies applied for 90 min at 37 °C. Immunodetection was performed with goat polyclonal anti-rabbit combined with Alexa Fluor 555 and chicken polyclonal anti-rat combined with Alexa Fluor 488 (A32732, A-21470, 1:200, Invitrogen, USA) antibodies incubated for 45 min at room temperature. Photos of the slides were taken with the Nikon Eclipse Ti and dedicated software (version 7.8.3). The photos were analyzed using a script written in the Matlab environment. Briefly, images in three channels (red—vessels, green—pericytes, blue—Hoechst) were enhanced for contrast, binarized using the Otsu method [83], and background subtracted. Additionally, an image of dilated microvessels was prepared (using the imdilate function) and the mask of pericytes near or collocated with the vessels was calculated on its basis. In the images prepared in this way, the microvessel density (the number of vessels per tumor slice), microvessel diameter (using the regionprops function on the red channel mask), pericyte coverage (the ratio of the pericyte signal near and colocalized with microvessels to the microvessel signal), Hoechst perfused area (expressed as a percent of the tumor section), and Hoechst leak (Hoechst intensity normalized to vessel density) were noted.

### 4.8. Ex Vivo Hypoxia and Invasiveness Assessment

Sections of harvested tumor tissue were used for the assessment of HIF-1a and vimentin expression with double immunofluorescence (IF) staining. The protocol of the staining and analysis was analogous to the one used with vascular morphology and perfusion assessment, but an additional step of 60 min of blocking with ReadyProbes™ Mouse-on-Mouse IgG Blocking Solution (R37621, Invitrogen, USA) was added before incubation with primary antibodies. Immunohistochemical staining was performed with mouse anti-HIF1a (MA1-16504, Invitrogen, 1:200) and rabbit anti-vimentin (SAB5700070, Sigma-Aldrich, 1:200) antibodies applied for 90 min at 37 °C. Immunodetection was performed with donkey polyclonal anti-rabbit combined with Alexa Fluor 555 and goat polyclonal anti-mouse combined with Alexa Fluor 488 (A32794, A11001, 1:200, Invitrogen, USA) antibodies incubated for 45 min in room temperature. Photos of the slides were taken with the Nikon Eclipse Ti and dedicated software (version 7.8.3). The photos were analyzed using a script written in the Matlab environment. After enhancing contrast, binarization, and background subtraction, the intensity and density of the stained area in each channel were calculated.

### 4.9. Enzyme-Linked Immunosorbent Assay (ELISA)

Parts of tumor tissue harvested at given endpoints were mechanically homogenized and centrifuged at 16,000× *g* and 4 °C for 10 min. The supernatant was collected, and the protein concentration of the lysate was determined by a BCA protein assay (Sigma-Aldrich, USA). Mouse VEGF-A levels in tumor tissue were measured using VEGF-A-Cell Lysate Mouse ELISA Kit (Invitrogen, USA) according to the manufacturer’s instructions. The samples were measured in triplicate.

### 4.10. Statistical Analysis

All results were presented as mean values and standard error of the mean (SEM). Statistical analysis was performed in STATISTICA 13.1 software (Stat-Soft Inc., Tulsa, OK, USA). For each parameter, the Shapiro–Wilk and Levene tests were used to determine a normal distribution and equality of variances, respectively. Depending on the data, one-way or two-way ANOVA followed by Tukey’s post-hoc HSD test was performed. P-values smaller than 0.05 were considered statistically significant.

## 5. Conclusions

In conclusion, we showed that both two-week metformin therapy and oxygen microbubble treatment normalize dysfunctional cancer vasculature, leading to less tortuous and leaky vasculature in a short time window. The combination of treatments led to longer and higher effects. All studied therapy protocols increased tumor oxygenation to 10–15 mm Hg, resulting in radiosensitization of 4T1 tumors and slower tumor growth after radiotherapy. However, resistance to treatment was inevitable, causing a fast return of hypoxia and increased metastasis.

## Data Availability

The data presented in this study are available on request from the corresponding author.
